# Validation and cross-cultural adaptation of the Turkish version of the revised Leeds Disability Questionnaire in patients with ankylosing spondylitis

**DOI:** 10.55730/1300-0144.5516

**Published:** 2022-07-24

**Authors:** Mehmet ÖZKESKİN, Fatih ÖZDEN, Özge OCAKER AKTAN, Tuba DEMİRCİ YILDIRIM, İsmail SARI

**Affiliations:** 1 Department of Physiotherapy and Rehabilitation, Faculty of Health Sciences, Ege University, İzmir, Turkey; 2 Department of Health Care Services, Köyceğiz Vocational School of Health Services, Muğla Sıtkı Koçman University, Muğla, Turkey; 3 Department of Physiotherapy and Rehabilitation, Institute of Health Sciences, Dokuz Eylül University, İzmir, Turkey; 4 Division of Rheumatology, Department of Internal Medicine, Faculty of Medicine, Dokuz Eylül University, İzmir, Turkey

**Keywords:** Ankylosing spondylitis, function, psychometrics, rheumatology

## Abstract

**Background/aim:**

The revised Leeds Disability Questionnaire (RLDQ) is a unique assessment tool for patients with ankylosing spondylitis (AS); its comprehensive structure includes posture and neck flexibility parameters. The aim of the study was to determine the psychometric properties of the Turkish RLDQ in patients with AS.

**Materials and methods:**

A total of 100 AS patients were enrolled in the study. In the first evaluation, patients filled out the Dougados Functional Index (DFI) and Bath Ankylosing Spondylitis Functional Index (BASFI), Stanford Health Assessment Questionnaire (HAQ) in addition to RLDQ. Then, patients were refilled the revised RLDQ in the second assessment.

**Results:**

The mean age of the patients (40 women, 60 men) was 48.3 ± 12.6 years. The test-retest reliability and internal consistency of the RLDQ total score were excellent. ICC score and Cronbach’s alpha score were calculated as 0.853 and 0.905, respectively. The SEM and MDC values calculated for the RLDQ total score were 2.74 and 7.60, respectively. RLDQ had degrees of correlation with DFI, HAQ, and BASFI of 0.814, 0.742, and 0.852, respectively. Construct validity was excellent (r > 0.50, p < 0.01).

**Conclusion:**

The Turkish version of the RLDQ was found to be valid and reliable in patients with AS. It should be emphasized that the RLDQ is a distinctive and valuable tool that focuses separately on neck, posture, or other mobility parameters in the clinical assessment of AS.

## 1. Introduction

Ankylosing spondylitis (AS) is a progressive, chronic inflammatory rheumatic disease characterized by pain, limitation of movement, and spinal deformity in general. Although spine involvement is more frequent, joint, extra-articular regions, and entheses are also affected. In individuals with AS, an increase in disability level and a decrease in quality of life are observed [1,2].

“Assessment of Spondylarthritis International Society-European League Against Rheumatism (ASAS/EULAR)” reported that combining nonpharmacological and pharmacological treatments is more effective in planning the treatment of individuals with AS [3]. The treatment approaches applied in patients with AS aim to reduce symptoms, provide spinal flexibility, preserve functionality, and increase health-related quality of life [4]. It is essential to evaluate the patient comprehensively before treatment. “The Assessment in Ankylosing Spondylitis Working Group” recommended some assessment tools for the determination of symptoms, disease severity, disease activity, spine mobility, and disability level while keeping clinical and physiotherapy records of patients [5]. In recent years, outcome measurements used in individuals with AS have been the focus of attention. Accordingly, the evaluations developed for patients with AS include disease status, clinical, physical, and functional evaluations, and patient-reported outcomes (PRO)’s [6]. PROs, which are used to evaluate chronic rheumatic diseases, deal with the effects of the disease on the patient in different dimensions [7]. Bath Ankylosing Spondylitis Functional Index (BASFI) [8], Ankylosing Spondylitis Disease Activity Score (ASDAS) [9], Patient Acceptable Symptom State (PASS) [10], and Ankylosing the Spondylitis Quality of Life (ASQoL) [11] are generally preferred for the function, disease activity, perceived general health status and quality of life, respectively. Core assessment tools recommended for the evaluation of the functional status of patients with AS include BASFI, Dougados Functional Index (DFI), Health Assessment Questionnaire for the Spondyloarthropathies (HAQ-S), The Revised Leeds Disability Questionnaire (RLDQ) [6, 12, 13]. BASFI and RLDQ are PRO’s [13], which evaluate functional status unidimensionally. Unlike other questionnaires, RLDQ is a short, understandable questionnaire developed more specifically for AS patients, including “mobility”, “bending down”, “reaching up and neck mobility”, and “posture”. Moreover, RLDQ includes the fields of “body functions”, “activities and participation”, “self-care” of the International Classification of Functioning, Disability, and Health (ICF) developed by the World Health Organization (WHO) [14]. The Swedish [15] and Italian [16] language versions of the RLDQ in individuals with AS were found to be valid and reliable. To our knowledge, the psychometric properties of the Turkish version of the RLDQ have not been studied yet. The aim of this study is to examine the cultural adaptation and psychometric properties of the Turkish version of The Revised Leeds Disability Questionnaire (RLDQ) in individuals with AS.

## 2. Methods

### 2.1. Translation and adaptation process

Necessary permissions were obtained from the original questionnaire developer to translate the RLDQ into Turkish and examine its psychometric properties. International methodological guidelines of Beaton et al. and Guillemin et al. were preferred for translation and cultural adaptation of the RLDQ [17,18]. The first step in the translation procedure is “forward-translation”. At this stage, an academic translation team consisting of four academics (one rheumatologist, three physiotherapists) whose mother tongue is Turkish and experts in English translated the questionnaire into Turkish independently and noted cultural and linguistic differences. The second step of the translation procedure is the “synthesis of translations”. The same four academic expert committee members discussed four independent translations in Turkish sociocultural and linguistic differences. Later, the Turkish draft of RLDQ was created. The third step of the translation procedure is the “back translation” of the synthesized translation (draft version). To check its accuracy, the draft translated version was back-translated from Turkish to English by a professional bilingual native English translator (without seeing the original version). At this stage, the aim is to find out whether the translated RLDQ represents linguistically the same content as the original version. In the fourth step of the translation procedure, the translation committee evaluated the conceptual compatibility of the RLDQ Turkish version. At this stage, conceptual and cultural aspects were discussed in detail. In the fifth step, RLDQ was piloted. The intelligibility, linguistic and cultural appropriateness of the RLDQ were analyzed with a 5-point Likert-type scale. A pretest was applied to 15 randomly selected Turkish-speaking healthy individuals. Since all the items were understandable in the pilot study, the latest version of the RLDQ was created without any changes (Appendix 1).

### 2.2. Sample size estimation

The sample size of the RLDQ was calculated separately for the total sample and test-retest reliability sample. The Kappa coefficient for the total score of the Revised LDQ in the Italian version was taken into account for the whole study [16]. The effect size calculated on this score (G*Power 3) was approximately 0.25 [19,20]. Considering the power of 0.80 and the probability of alpha error of 0.05, it was appropriate to conduct the study with a total of 95 individuals. Considering the possible drop-out rate, we completed the study with 100 individuals. Additionally, calculation formulas and recommendations of Walter and Bonett were taken into account for test-retest reliability [21,22]. The lower interval of the ICC value in the revised LDQ psychometric property analysis study was determined as (0.94) expected and 0.80 as the least acceptable ICC. Alpha significance level and power were adjusted as 0.05 and 0.80, respectively [13]. Considering the 10% drop-out, it was decided that at least 24 individuals should be reevaluated. Consequently, 30 individuals were retested.

### 2.3. Study design

The study was conducted with 100 AS patients who were followed up at Dokuz Eylül University, Department of Rheumatology. Inclusion criteria for the study were patients diagnosed with AS according to the modified New York criteria (mNYc) by a rheumatologist were included in the study [23].

Exclusion criteria for the study were individuals with other chronic and severe conditions (e.g., hemiplegia, neuropathies, lumbar disc herniations, spinal pathologies). An informed consent form was signed by the patients and the study was approved by the ethics committee of Ege University (No: 21-5.1T/4).

The physical data and demographic characteristics of the patients were assessed. Patients were asked to fill the Dougados Functional Index (DFI) and Bath Ankylosing Spondylitis Functional Index (BASFI), Stanford Health Assessment Questionnaire (HAQ) in addition to RLDQ. Thirty patients refilled the RLDQ [24,25].

#### Revised Leeds Disability Questionnaire (RLDQ)

The Revised Leeds Disability Questionnaire (RLDQ) consists of four main sections and a total of 16 items. Each section contains 4 items: “mobility”, “bending down”, “reaching up and neck mobility”, and “posture”. Each item is scored from 0 (I can do it without difficulty) to 3 (I cannot). The originally proposed procedure for calculating the total score has been changed. Currently, it is recommended that the sum of the scores obtained from each of the 16 items constitute a total score ranging from 0 to 48 [13].

#### Dougados Functional Index (DFI)

DFI is a 20-question survey of difficulties in doing daily activities. This tool is the index of functional decline, and it is used for scoring joint tenderness in AS assessment. The functional index consists of 20 questions, and the joint index is based on the scoring of 10 joints after strong finger pressure. The Turkish version of the questionnaire was found to be valid and reliable [24].

#### Bath Ankylosing Spondylitis Functional Index (BASFI)

BASFI consists of 10 questions in total, including functional activities related to reaching, bending, changing positions, getting up, turning and climbing stairs (eight questions) and the ability of patients to cope with their daily lives (two questions). Patients answer the questions on the Visual Analog Scale (VAS). BASFI questions the physical functions of the patients in the last week. A score between 0–10 is obtained from the scale. An increase in the score indicates an increase in physical function limitation. The Turkish version of the questionnaire was found to be valid and reliable [24].

#### Stanford Health Assessment Questionnaire (HAQ)

The Stanford Health Assessment Questionnaire (HAQ) is an arthritis-specific scale for assessing functional status. It is widely used in patients with AS. HAQ is a questionnaire reflecting functional status, and its score has been shown to correlate with disease activity indicators. HAQ scores range from 0 to 3, with 3 points indicating severe functional disability. HAQ has eight domains. There are 20 questions in total, 2–3 questions in each area. These areas are; dressing and self-care, getting up, eating, walking, hygiene, reaching, grasping, and normal daily activities. While filling out the questionnaire, the last week is questioned. The patient’s difficulties in certain activities are evaluated. The Turkish version of the questionnaire was found to be valid and reliable [25].

### 2.4. Statistical analysis

“IBM SPSS Statistics Version 25 computer package program (Chicago, IBM, USA)” was preferred for data analysis. “Mean ± standard deviation (X±SD)” or “%” was given for the quantitative and qualitative variables, respectively. “Kolmogorov-Smirnov/Shapiro-Wilk” tests were analyzed to check the normality. “The intra-class correlation coefficient (ICC)” was used to evaluate “test-retest reliability”. ICC values greater than 0.80 was accepted as excellent reliability (26). “Internal consistency” was analyzed with “Cronbach’s alpha coefficient”. Alpha values ranged 0.70 and 0.95 were defined as a consistent score [27]. In addition, standard error mean-standard error mean (SEM_95_) and minimum detectable change-minimal detectable change (MDC_95_) were also calculated [28]. The relationship between RLDQ and other questionnaires was analyzed for construct validity. The statistical significance level was accepted as p < 0.05. The confidence interval was set as 0.05. Juniper emphasized that a correlation coefficient above 0.5 show an excellent validity of a questionnaire [29].

## 3. Results

The mean age of 100 patients (40 female, 60 male) participating in the study was 48.3 ± 12.6 years. The participant’s body mass index was 27.3 ± 5.2 kg/m2, classified as slightly overweight. The mean time elapsed after individuals were diagnosed with AS was 12.4 ± 9.1. The majority of the patients were married (83%). On the other hand, 41% and 33% of individuals were high school and primary school graduates, respectively. Sociodemographic variable data of the patients are presented in Table 1.

The values of DFI HAQ, BASFI, and RLDQ measurements were 8.5 ± 6.8, 11.2 ± 13.5, 3.6 ± 2.5, and 10.4 ± 8.9, respectively (Table 2). The test-retest reliability and internal consistency of the RLDQ total score were excellent. ICC score was calculated as 0.853, and Cronbach’s alpha score was calculated as 0.905. The reproducibility of the items was between 0.6 and 0.8. That is, high to excellent reliability was recorded. In addition, the internal consistency score of the total score and the items was between 0.80 and 0.9. This value confirmed the internal consistency of the RLDQ (Table 3).

The SEM and MDC values calculated for the RLDQ total score were 2.74 and 7.60, respectively. On the other hand, within the scope of construct validity, LDQ; DFI was compared with HAQ and BASFI. The correlation coefficients obtained from this comparison ranged from 0.7 to 0.8. RLDQ had degrees of correlation with DFI, HAQ, and BASFI of 0.814, 0.742, and 0.852, respectively. These r values revealed that the RLDQ was the valid assessment score for disability and quality of life. Construct validity was excellent (r > 0.50, p < 0.01) (Table 4).

## 4. Discussion

The present study focused on the cultural adaptation, validity, and reliability of the Turkish version of the RLDQ. This straightforward questionnaire is frequently used by rheumatologists in patients with AS, developed more than 25 years ago [30]. During the rehabilitation process, the focus of physiotherapists on daily living activities for specific movements raises the need for a questionnaire containing subheadings for each restricted movement, joint and posture [31]. In this respect, the RLDQ has provided a unique assessment chance to therapists. It was essential to translate and adapt the questionnaire with standardized methods. This procedure provides a reliable evaluation in individuals whose mother tongue is Turkish, living in Turkey or in various locations in Europe [32]. According to the study results, the Turkish version of the RLDQ was found to be valid and reliable.

To emphasize the unique advantage of our research, it is necessary to discuss the superiority of the RLDQ over similar assessment tools. The RLDQ consists of 4 subitems and a total of 16 questions. These subitems are; “mobility”, “bending down”, “reaching up and neck mobility”, and “posture”. In this respect, it differs from the disability assessment in other ankylosing spondylitis. Physiotherapists need to be able to evaluate certain body parts, joints, movements and posture, independently. Being able to do this with a standardized questionnaire adds great specificity to clinical practice. For instance, posture can be evaluated comprehensively in the RLDQ. It is essential for patients to be able to express their difficulties comprehensively in cases of standing on the heel, coughing-sneezing, sleeping in the supine or prone position [13, 16,30]. In addition, limitations due to decreased flexibility in the neck joint are prevalent in AS. In rehabilitation follow-up, patients’ range of motion is usually monitored with a goniometer or inclinometer [33]. However, besides objective findings, presenting the patient’s subjective difficulty in neck movements by blending them with daily living activities may provide clinicians with an additional advantage in evaluation. BASFI does not provide a comprehensive assessment with subsections. In addition, the ceiling effect has been reported in BASFI. This issue shows that the actual scores of the patients reflected commonly high with BASFI. It is deemed that BASFI may not be responsive enough to reveal patients’ clinical status [6,12,34]. The Dougados Function Index is another frequently used questionnaire. Although it can evaluate comprehensive activities of daily living, it has a narrow response range in terms of giving answers (Likert type ranged 0–2). In this case, it may lead to patients not being able to express their own situation adequately. In addition, subscores may not allow the physiotherapist to make a comprehensive evaluation [12,34]. Therefore, considering that different questionnaires may provide different benefits, clinicians may focus on the RLDQ, especially in order to address posture and neck joint mobility individually.

The LDQ was developed in 1994 and later revised [13,30]. The Italian and Swedish versions of the adapted questionnaire were found to be valid and reliable [15,16]. We could not find another language version of this questionnaire, which is frequently used in ankylosing spondylitis, other than English, Swedish and Italian, or perhaps it was not published in an article. However, it should be emphasized that this is a significant deficiency and that rheumatological study groups should use this questionnaire by revealing its psychometric properties in different countries [27].

In the original English development study (sample age 41.1 and 46.3 years), the RLDQ was found to be responsive between the two assessments (t = 2.79, p < 0.01). In this development study, whose sample age was similar to our study (approximately 48 years), ICC was presented as 0.92. In the correlation results, the authors showed a wide range of coefficients from low to high (0.2 to 0.7) in correlation with some clinical examination findings of flexibility (e.g., Schober’s test, flexibility assessment) [30]. In the later study of revised LDQ, responsiveness (p < 0.0001) and reliability (ICC = 0.95) were also revealed [13].

In the Italian version of the RLDQ, Cronbach’s alpha value of the questionnaire was calculated and found to be 0.90. The ICC was 0.97, and the correlation coefficient between HAQ and Italian RLDQ was 0.8, considered remarkable. It has been shown that the correlation with anthropometric measurements is between 0.2–0.7 in the Italian version, similar to the development study [16]. In the Swedish version, internal consistency and test-retest reliability were demonstrated by correlation and kappa coefficient, and the questionnaire was reliable. On the other hand, in the correlational analysis performed for construct validity, a correlation was obtained between the anthropometric measurements and the RLDQ in the range of 0.00–0.64 [15].

In our study, the Turkish version of the RLDQ revealed an ICC value of 0.85. Although it is a slightly low value compared to other psychometric analysis studies, being >0.80 showed that the test-retest reliability of the Turkish version of the questionnaire was high. We also showed excellent internal consistency with an alpha value (α = 0.905). The SEM and MDC value of the RLDQ was demonstrated for the first time in our study. In this respect, our study has a unique originality. The MDC value of 7.60 provides a reference follow-up value for rheumatologists and therapists in clinical practice. The smallest change value that can be considered clinically significant has been demonstrated.

We examined the construct validity of the Turkish RLDQ with correlational analysis. The correlation between the RLDQ and HAQ was calculated as 0.74. This value was close to the HAQ comparison coefficient in the Italian version (0.80) [16]. In this respect, our study was similar to the Italian version. In addition, the correlations between RLDQ with DFI and BASFI were 0.81 and 0.85, respectively. In our study, these correlation values indicated a high degree of correlation of the Turkish version of the RLDQ.

Revealing some limitations of our study will contribute to the methods of further studies. First of all, we did not compare the RLDQ with any anthropometric features of the patients. In particular, joint mobility measurement with goniometer and inclinometer or comparison with a more precise objective measurement would have made our study more valuable. Alternatively, evaluation of flexibility with clinical tests (e.g., Schober test, sit and reach test) could provide us with an advantageous clinical conclusion. Second, we did not do a responsiveness analysis. This analysis method was not suitable for the specifications of our study, as it required long-term patient follow-up or investigated the response to treatment intervention. Third, we did not do the content validity of the revised LDQ. In this way, further adaptation could be obtained. Last but not least, a comprehensive postural assessment of patients, particularly by a sensor or computerized analysis, would have been of additional advantage compared to the LDQ.

## 5. Conclusions

According to the results of our study, the Turkish version of the RLDQ was found to be valid and reliable in patients with ankylosing spondylitis. Test-retest reliability and internal consistency were excellent, and construct validity was high. It has been shown that the RLDQ has a high correlation coefficient with the disability assessment questionnaires that are frequently used in the field. On the other hand, the most original aspect of the study was that the SEM and MDC values of the Revised LDQ were revealed for the first time. It should be emphasized that the revised LDQ is distinctive and valuable to focus separately on neck mobility and posture, individually.

## Figures and Tables

**Table t5-turkjmedsci-52-5-1729:** 

	Zorluk çekmeden yapabilirim	Zorlanarak yapabilirim	Sadece olması gerekenin dışında ekstra hareketler ile yapabilirim	Yapamam
**1. HAREKETLİLİK** a. Banyoya girip çıkma				
b. Arabaya binme ve inme				
c. Sabah uyanıp yataktan kalkma				
d. Yatakta dönme				
**2. AŞAĞI EĞİLME** a. Tuvaleti kullandıktan sonra temizlenme				
b. Çorap giyme ve çıkarma				
c. Ayakkabı giyme ve bağcıklarını bağlama				
d. Ayak tırnaklarını kesme				
**3. BOYUN** hareketleri a. Yüksekteki pencereleri açma				
b. Yoldan karşıya geçmeden önce iki yöne de bakma (örneğin bakmak için ayaklarınızı hareket ettirmeniz gerekiyor mu?)				
c. Yüksek bir rafa uzanırken ulaştığınız objeye bakma				
d. Küçük bir bardaktan içecek içme (örneğin dizinizi bükmek zorunda kalıyor musunuz?)				
**4. DURUŞ** a. Topukların üzerinde yürüme				
b. Öksürme veya hapşırma				
c. Sırt üstü uyuma				
d. Yüzüstü uyuma				

**Table 1:** The data of the ankylosing spondylitis patients.

n:100	Total
Age (years, mean ± SD)	48.3 ± 12.6
Weight (kg, mean ± SD)	78.1 ± 15.2
Height (cm, mean ± SD)	1.69 ± 0.09
Body mass index (kg/m^2^)	27.3 ± 5.2
Duration of AS (years, mean ± SD)	12.4 ± 9.1
Gender (n, %)	
Women	40 (40.0)
Men	60 (60.0)
Marital status (n, %)	
Married	83 (83.0)
Single	17 (17.0)
Education (n, %)	
Primary school	33 (33.0)
High school	41 (41.0)
University	24 (24.0)
Postgraduate	2 (2.0)

SD: standard deviation, n: number of patients

**Table 2:** The mean clinical scores of the ankylosing spondylitis patients.

n:100	Mean ± SD	Range
DFI	8.5 ± 6.8	(0–27)
HAQ	11.2 ± 13.5	(0–100)
BASFI	3.6 ± 2.5	(0–5)
RLDQ	10.4 ± 8.9	(0–38)

SD: standard deviation, n: number of patients

**Table 3:** The reliability of the RLDQ.

	Test (Mean ± SD)	Retest (Mean±SD)	ICC (95% CI)	α	SEM_95_	MDC_95_
Item 1a	0.3 ± 0.5	0.3 ± 0.6	0.726 (0.42–0.86)	0.898	0.21	0.60
Item 1b	0.4 ± 0.6	0.2 ± 0.5	0.809 (0.59–0.90)	0.899	0.24	0.68
Item 1c	0.9 ± 0.8	0.9 ± 0.6	0.828 (0.63–0.91)	0.902	0.37	1.04
Item 1d	0.5 ± 0.6	0.5 ± 0.6	0.777 (0.53–0.89)	0.898	0.24	0.66
Item 2a	0.3 ± 0.6	0.4 ± 0.6	0.838 (0.66–0.92)	0.900	0.24	0.68
Item 2b	0.9 ± 0.9	0.9 ± 1.0	0.832 (0.64–0.92)	0.896	0.52	1.44
Item 2c	0.9 ± 0.1	0.8 ± 0.8	0.664 (0.29–0.84)	0.892	0.04	0.12
Item 2d	1.1 ± 1.0	1.1 ± 1.0	0.789 (0.55–0.89)	0.894	0.36	0.99
Item 3a	0.5 ± 0.8	0.4 ± 0.8	0.870 (0.72–0.93)	0.901	0.26	0.73
Item 3b	0.8 ± 0.9	0.7 ± 0.8	0.890 (0.76–0.94)	0.901	0.33	0.93
Item 3c	0.6 ± 0.8	0.6 ± 0.8	0.859 (0.70–0.93)	0.901	0.27	0.75
Item 3d	0.3 ± 0.6	0.7 ± 1.0	0.884 (0.75–0.94)	0.899	0.30	0.84
Item 4a	0.7 ± 1.0	0.4 ± 0.7	0.744 (0.46–0.87)	0.897	0.46	1.29
Item 4b	0.1 ± 0.5	0.7 ± 1.1	0.782 (0.54–0.89)	0.898	0.20	0.57
Item 4c	0.7 ± 1.0	0.7 ± 1.0	0.828 (0.63–0.91)	0.903	0.54	1.51
Item 4d	1.0 ± 1.3	0.8 ± 1.1	0.703 (0.37–0.85)	0.905	0.49	1.38
**RLDQ (T)**	10.6 ± 8.9	9.6 ± 8.2	0.853 (0.69–0.93)	0.905	2.74	7.60

n: number of patients, ICC: Intra-class correlation coefficient, CI: Confidence interval, α: Cronbach’s alpha, SEM: Standard error of measurement; MDC: Minimal detectable change.

**Table 4:** Correlation between the RLDQ with DFI, HAQ, BASFI.

n:100	r	p
**RLDQ vs DFI**	0.814	<0.01
**RLDQ vs HAQ**	0.742	<0.01
**RLDQ vs BASFI**	0.852	<0.01

n: number of patients

## Leeds Yetersizlik Anketi

LÜTFEN BU KAYDI DOLDURUN VE BUGÜN SİZİ MUAYENE EDEN DOKTORA VERİN.

LÜTFEN **GE****Ç****EN HAFTA** BOYUNCA YETENEKLERİNİZİ EN İYİ AÇIKLAYAN YANITI İŞARETLEYİN.

[Table t5-turkjmedsci-52-5-1729]LÜTFEN SÜTUN 3’E DİKKAT EDİN (OLMASI GEREKENİN DIŞINDAKİ EKSTRA HAREKETLER VURGULANIYOR): Örneğin, eğer arabadan ancak elinizi yukarı kaldırıp tavandan destek alarak inebiliyorsanız, soru 1b’ye yanıt olarak bu sütunu işaretleyebilirsiniz.

## References

[1] Oskay D Tuna Z Düzgün İ Elbasan B Yakut Y Relationship between kinesiophobia and pain, quality of life, functional status, disease activity, mobility, and depression in patients with ankylosing spondylitis Turkish Journal of Medical Sciences 2017 47 5 1340 1347 10.3906/sag-1702-93 29151302

[2] Baykara RA Küçük A Tuzcu A Tuzcu G Cüre E The relationship of serum visfatin levels with clinical parameters, flow-mediated dilation, and carotid intima-media thickness in patients with ankylosing spondylitis Turkish Journal of Medical Sciences 2021 51 4 1865 1874 10.3906/sag-2012-351 33754654 PMC8569753

[3] Zochling J Van Der Heijde D Burgos Vargas R Collantes E Davis JC ASAS/EULAR recommendations for the management of ankylosing spondylitis Annals of the Rheumatic Diseases 2006 65 4 442 452 10.1136/ard.2005.041137 16126791 PMC1798102

[4] Chiowchanwisawakit P Thaweeratthakul P Wattanamongkolsil L Srinonprasert V Koolvisoot A Relationship between health-related quality of life and patient acceptable symptom state with disease activity and functional status in patients with ankylosing spondylitis in Thailand Journal of Clinical Rheumatology 2019 25 1 16 23 10.1097/RHU.0000000000000750 29509563

[5] Van Der Heijde D Calin A Dougados M Khan M Van Der Linden S Selection of instruments in the core set for DC-ART, SMARD, physical therapy, and clinical record keeping in ankylosing spondylitis. progress report of the ASAS working group. assessments in ankylosing spondylitis The Journal of Rheumatology 1999 26 4 951 954 10.3899/jrheum.211322 10229426

[6] Zochling J Measures of symptoms and disease status in ankylosing spondylitis: ankylosing spondylitis disease activity score (ASDAS), ankylosing spondylitis quality of life scale (ASQoL), bath ankylosing spondylitis disease activity index (BASDAI), bath ankylosing spondylitis functional index (BASFI), bath ankylosing spondylitis global score (BAS-G), bath ankylosing spondylitis metrology index (BASMI), dougados functional index (DFI), and health assessment questionnaire for the spondylarthropathies (HAQ-S) Arthritis Care & Research 2011 63 S11 47 58 10.1002/acr.20575 22588768

[7] Rodrigues Manica S Silva J Cruz Machado R Coelho C Duarte J Biologic disease-modifying anti-rheumatic drugs and patient-reported outcomes in axial SpA: a systematic review and a call for action Clinical Rheumatology 2021 40 1 33 41 10.1007/s10067-020-05209-x 32533340

[8] Calin A Garrett S Whitelock H Kennedy LG O’hea J A new approach to defining functional ability in ankylosing spondylitis: the development of the bath ankylosing spondylitis functional index The Journal of Rheumatology 1994 21 12 2281 2285 https://doi.org/780429218316 7699629

[9] Machado P Landewé R Lie E Kvien TK Braun J ankylosing spondylitis disease activity score (ASDAS): defining cut-off values for disease activity states and improvement scores Annals of the Rheumatic Diseases 2011 70 1 47 53 https://doi.org/ard.2010.138594 21068095 10.1136/ard.2010.138594

[10] Maksymowych WP Richardson R Mallon C Van Der Heijde D Boonen A Evaluation and validation of the patient acceptable symptom state (PASS) in patients with ankylosing spondylitis Arthritis and Rheumatism 2007 57 1 133 139 10.1002/art.22469 17266072

[11] Doward L Spoorenberg A Cook S Whalley D Helliwell P Development of the ASQoL: a quality of life instrument specific to ankylosing spondylitis Annals of the Rheumatic Diseases 2003 62 1 20 26 10.1136/ard.62.1.20 12480664 PMC1754293

[12] Cardiel M Londono J Gutiérrez E Pacheco Tena C Vázquez Mellado J Translation, cross-cultural adaptation, and validation of the bath ankylosing spondylitis functional index (basfi), the bath ankylosing spondylitis disease activity index (BASDAI) and the dougados functional index (DFI) in a Spanish speaking population with spondyloarthropathies Clinical and Experimental Rheumatology 2003 21 4 451 458 12942696

[13] Eyres S Tennant A Kay L Waxman R Helliwell PS Measuring disability in ankylosing spondylitis: comparison of bath ankylosing spondylitis functional index with revised leeds disability questionnaire The Journal of Rheumatology 2002 29 5 979 986 12022361

[14] Sigl T Cieza A Van Der Heijde D Stucki G ICF based comparison of disease specific instruments measuring physical functional ability in ankylosing spondylitis Annals of the Rheumatic Diseases 2005 64 11 1576 1581 10.1136/ard.2004.027185 15843457 PMC1755279

[15] Stenström CH Hellström S Hultgren M Wikström M Reliability and validity of a swedish version of the revised leeds disability questionnaire for patients with ankylosing spondylitis Scandinavian Journal of Rheumatology 2000 29 4 243 248 10.1080/030097400750041398 11028846

[16] Lubrano E Sarzi Puttini P Parsons W D’Angelo S Cimmino M Validity and reliability of an Italian version of the revised leeds disability questionnaire for patients with ankylosing spondylitis Rheumatology (Oxford) 2005 44 5 666 669 10.1093/rheumatology/keh578 15757970

[17] Beaton DE Bombardier C Guillemin F Ferraz MB Guidelines for the process of cross-cultural adaptation of self-report measures Spine 2000 25 24 3186 3191 10.1097/00007632-200012150-00014 11124735

[18] Guillemin F Bombardier C Beaton D Cross-cultural adaptation of health-related quality of life measures: literature review and proposed guidelines Journal of Clinical Epidemiology 1993 46 12 1417 1432 10.1016/0895-4356(93)90142-N 8263569

[19] Faul F Erdfelder E Buchner A Lang AG Statistical power analyses using G* Power 3.1: Tests for correlation and regression analyses Behavior Research Methods 2009 41 4 1149 1160 10.3758/BRM.41.4.1149 19897823

[20] Faul F Erdfelder E Lang AG Buchner A G* Power 3: A flexible statistical power analysis program for the social, behavioral, and biomedical sciences Behavior Research Methods 2007 39 2 175 191 10.3758/BF03193146 17695343

[21] Walter S Eliasziw M Donner A Sample size and optimal designs for reliability studies Statistics in Medicine 1998 17 1 101 110 10.1002/(SICI)1097-0258(19980115)17:1<101::AID-SIM727>3.0.CO;2-E 9463853

[22] Bonett DG Sample size requirements for estimating intraclass correlations with desired precision Statistics in Medicine 2002 21 9 1331 1335 10.1002/sim.1108 12111881

[23] Chung HY Lau CS Wu KP Wong WS Mok MY Comparison of performance of the assessment of spondyloarthritis international society, the european spondyloarthropathy study group and the modified new york criteria in a cohort of chinese patients with spondyloarthritis Clinical Rheumatology 2011 30 7 947 953 10.1007/s10067-011-1693-6 21336823 PMC3123457

[24] Karatepe AG Akkoc Y Akar S Kirazli Y Akkoc N The Turkish versions of the bath ankylosing spondylitis and dougados functional indices: reliability and validity Rheumatology international 2005 25 8 612 618 10.1007/s00296-004-0481-x 15248085

[25] Küçükdeveci AA Sahin H Ataman S Griffiths B Tennant A Issues in cross‐cultural validity: example from the adaptation, reliability, and validity testing of a Turkish version of the stanford health assessment questionnaire Arthritis Care & Research 2004 51 1 14 19 10.1002/art.20091 14872450

[26] Baumgartner TA Chung H Confidence limits for intraclass reliability coefficients Measurement in Physical Education and Exercise Science https://doi.org/2001;5(3):179-88 10.1207/S15327841MPEE0503_4

[27] Terwee CB Bot SD De Boer MR Van Der Windt DA Knol DL Quality criteria were proposed for measurement properties of health status questionnaires Journal of Clinical Epidemiology 2007 60 1 34 42 10.1016/j.jclinepi.2006.03.012 17161752

[28] Portney LG Watkins MP Foundations of Clinical Research: Applications to Practice 3rd ed NJ, USA Pearson/Prentice Hall Upper Saddle River 2009

[29] Juniper EF Guyatt GH Jaeschke R How to develop and validate a new health-related quality of life instrument Spilker B Quality of Life and Pharmacoeconomics in Clinical Trials 2nd ed Philadelphia, USA Lippincott-Raven Publishers 1996 49 56

[30] Abbott C Helliwell P Chamberlain M Functional assessment in ankylosing spondylitis: evaluation of a new self-administered questionnaire and correlation with anthropometric variables Rheumatology 1994 33 11 1060 1061 https://doi.org10.1093/rheumatology/33.11.1060 10.1093/rheumatology/33.11.1060 7981994

[31] Haywood K Garratt A Jordan K Dziedzic K Dawes P Spinal mobility in ankylosing spondylitis: reliability, validity and responsiveness Rheumatology 2004 43 6 750 757 10.1093/rheumatology/keh169 15163832

[32] Özden F Tuğay N Tuğay BU Kılınç CY Psychometrical properties of the Turkish translation of the new knee society scoring system Acta Orthopaedica et Traumatologica Turcica 2019 53 3 184 188 10.1016/j.aott.2019.03.003 30961927 PMC6601126

[33] Maksymowych WP Mallon C Richardson R Conner-Spady B Jauregui E Development and validation of a simple tape-based measurement tool for recording cervical rotation in patients with ankylosing spondylitis: comparison with a goniometer-based approach The Journal of Rheumatology 2006 33 11 2242 2249 17013998

[34] Moncur C Ankylosing spondylitis measures: The ankylosing spondylitis quality of life (ASQOL) scale, bath ankylosing spondylitis disease activity index (BASDAI), bath ankylosing spondylitis functional index (BASFI), bath ankylosing spondylitis global score (BAS-G), bath ankylosing spondylitis metrology index (BASMI), dougados functional index (DFI), health assessment questionnaire for the spondyloarthropathies (HAQ-S), and revised leeds disability questionnaire (RLDQ) Arthritis Care & Research: Official Journal of the American College of Rheumatology 2003 49 5 197 209 10.1002/art.11412 22588768

